# Persistent Sepsis-Induced Hypotension without Hyperlactatemia: A Distinct Clinical and Physiological Profile within the Spectrum of Septic Shock

**DOI:** 10.1155/2012/536852

**Published:** 2012-04-18

**Authors:** Glenn Hernandez, Alejandro Bruhn, Ricardo Castro, Cesar Pedreros, Maximiliano Rovegno, Eduardo Kattan, Enrique Veas, Andrea Fuentealba, Tomas Regueira, Carolina Ruiz, Can Ince

**Affiliations:** ^1^Department of Translational Physiology, Academic Medical Center, University of Amsterdam, Meibergdreef 9, 1105 AZ Amsterdam, The Netherlands; ^2^Departamento de Medicina Intensiva, Pontificia Universidad Católica de Chile, Marcoleta 367, 8320000 Santiago, Chile

## Abstract

*Introduction*. A subgroup of septic shock patients will never develop hyperlactatemia despite being subjected to a massive circulatory stress. Maintenance of normal lactate levels during septic shock is of great clinical and physiological interest. Our aim was to describe the clinical, hemodynamic, perfusion, and microcirculatory profiles associated to the absence of hyperlactatemia during septic shock resuscitation. *Methods*. We conducted an observational study in septic shock patients undergoing resuscitation. Serial clinical, hemodynamic, and perfusion parameters were registered. A single sublingual microcirculatory assessment was performed in a subgroup. Patients evolving with versus without hyperlactatemia were compared. *Results*. 124 septic shock patients were included. Patients without hyperlactatemia exhibited lower severity scores and mortality. They also presented higher platelet counts and required less intensive treatment. Microcirculation was assessed in 45 patients. Patients without hyperlactatemia presented higher PPV and MFI values. Lactate was correlated to several microcirculatory parameters. No difference in systemic flow parameters was observed. *Conclusion*. Persistent sepsis-induced hypotension without hyperlactatemia is associated with less organ dysfunctions and a very low mortality risk. Patients without hyperlactatemia exhibit less coagulation and microcirculatory derangements despite comparable macrohemodynamics. Our study supports the notion that persistent sepsis-induced hypotension without hyperlactatemia exhibits a distinctive clinical and physiological profile.

## 1. Introduction

Although the physiologic basis of lactate generation during shock has been recently matter of debate and research, a perfusion-related mechanism is probably involved at least in early stages [1–3]. Recent clinical studies have confirmed the strong prognostic value of hyperlactatemia and its association to other hemodynamic and perfusion abnormalities in septic shock [4–6]. Either a single abnormal level or an impaired lactate clearance is related to morbidity and mortality.

More intriguingly, a subgroup of septic patients requiring prolonged vasopressor support, and thus classified as septic shock according to the 2001 Sepsis Definition Conference [7], will never develop hyperlactatemia despite being subjected to a massive circulatory stress [8, 9]. Moreover, we recently performed a retrospective analysis of 302 vasopressor-requiring septic patients, and demonstrated that the absence of hyperlactatemia was associated with a very low (7.7%) mortality risk as compared with that in patients presenting hyperlactatemia at some point during resuscitation (42%) [9].

The maintenance of normal lactate levels in a septic patient with circulatory dysfunction is of great clinical and physiological interest. In fact, since several potential mechanisms can induce hyperlactatemia, including low cardiac output, microcirculatory abnormalities, sustained hyperadrenergia with accelerated aerobic glycolysis, and hepatosplanchnic hypoperfusion, among others, it is likely that the absence of hyperlactatemia reflects a more adequate physiological response to stress [1, 9]. Indeed, the very low mortality associated to this condition supports the notion of a relatively preserved global homeostasis [9]. However, this statement is highly speculative and should be addressed in additional clinical and physiological research specifically focused on the determinants of lactate homeostasis during sepsis-related circulatory dysfunction.

Our aim was to describe the clinical, hemodynamic, perfusion, and microcirculatory profiles associated to the absence of hyperlactatemia during septic shock resuscitation as a hypothesis-generating study.

## 2. Patients and Methods

We conducted an observational study from April 2008 to October 2010, including all adult patients admitted to the ICU with a diagnosis of septic shock according to the 2001 Sepsis Definition Conference [7]. Under this definition, septic patients are considered in shock when presenting a volume-refractory hypotension and thus require vasopressors to sustain blood pressure.

All septic shock patients were treated with a periodically updated management protocol independently of their participation in this study, and their demographic and clinical data were registered in a prospective data set. The Institutional Review Board (IRB) of our University approved this study and waived the necessity of an informed consent because of the solely observational nature of the study design, and considering that it did not deviate from the best standard of care.

Patients requiring vasopressors to maintain mean arterial pressure (MAP) > 65 mmHg despite initial fluid loading [10] and committed to full resuscitation were considered eligible for this study.

Our local management algorithm for septic shock has been published elsewhere [9, 11–14]. Septic patients presenting a circulatory dysfunction at the emergency department (ED) or the pre-ICU service were subjected to vigorous fluid resuscitation and basal measurements of lactate (Radiometer ABL 735, Copenhagen Denmark). If developing persistent hypotension or hyperlactatemia, patients were transferred to the ICU as soon as a bed was available. In the meantime, and depending on the timing of ICU bed availability, a central venous catheter was inserted for measurement of central venous oxygen saturation. The mean transfer time from the ED to the ICU for septic shock patients in our university hospital is 48 minutes [14].

ICU-based resuscitation was aimed at normalizing macrohemodynamic and clinical and metabolic perfusion parameters. Invasive hemodynamic monitoring and mechanical ventilation (MV) were decided on an individual basis by attending physicians. Norepinephrine (NE) was used as the sole vasopressor and adjusted to the minimal dose to maintain the MAP target. Optimal fluid resuscitation was guided by dynamic predictors [15] or by a Starling curve approach when the former were not feasible. High-volume hemofiltration (HVHF) was indicated as a final salvage therapy in unresponsive patients [13]. Intra-abdominal pressure was monitored and treated according to recent guidelines [16]. Complementarily, a dedicated sepsis team performed a daily exhaustive reassessment of the adequacy of source control and participated in major decisions.

Perfusion assessment included metabolic (arterial lactate, central (ScvO_2_) or mixed (SvO_2_) venous O_2_ saturation, central venous-to-arterial PCO_2_ difference (P(cv-a)CO_2_)) and peripheral perfusion parameters (capillary refill time, central-to-peripheral temperature gradient, skin mottling) at least every 6 h during the first 48 h of treatment. A patient with septic shock was subjected to at least 9 arterial lactate determinations (including the first pre-ICU assessment) during this period.

A patient was considered as resuscitated when normalization of both metabolic and peripheral perfusion parameters was achieved, while maintaining stable or decreasing NE requirements for at least 12 h. Patients were followed until hospital discharge or death. Baseline values were registered after arterial line and central venous catheter insertion. Sublingual microcirculatory assessments were performed within 6 h of ICU resuscitation in a subgroup of patients (see below).

We divided the whole cohort according to the presence or not of any abnormal lactate value during the resuscitation period and compared the resulting subgroups for differences in mortality and other relevant clinical and physiological variables. To be classified to the “normal” lactate subgroup, all lactate measurements including the pre-ICU determinations had to be in the normal range. Patients with at least one abnormal level were classified to the “hyperlactatemia” subgroup.

## 3. Lactate Determination

Lactate levels were measured in arterial blood using the hospital's central laboratory through a blood gas analyzer (Radiometer ABL 735, Copenhagen, Denmark). According to our laboratory standards, a range from 0.1 to 2.4 mmol/L was considered as normal. This cut-off was recently revalidated by Shapiro et al. [4].

### 3.1. Sublingual Microcirculation Imaging

Microcirculatory assessments were performed in all septic shock patients included after April 2010. At this point, proper training of staff in image acquisition was completed, thus allowing around-the-clock availability. A different investigator, who was blinded to clinical data, performed image analysis according to a recent consensus [17].

Sublingual microcirculation was assessed with sidestream dark field (SDF) videomicroscopy with a 5x lens (Microscan for NTSC, Microvision Medical). At each time point, at least five 10–20 sec images were recorded. After removing saliva and oral secretions, the probe was applied over the mucosa at the base of the tongue. Special care was taken to avoid exerting excessive pressure on the mucosa, which was verified by checking ongoing flow in larger microvessels (>50 um). Analog images were digitalized by using the pass-through function of a digital video camera recorder (Sony DCR-HC96, for NTSC) and were recorded instantaneously and transformed to AVI format in a laptop with the aid of a commercial software (DVGate Plus 2.3, Sony Corporation).

According to recommendations of the cited consensus [17], image analysis consisted in flow (percentage of perfused vessels, PPV; microcirculatory blood flow, MFI), density (total vascular density, TVD; perfused vascular density, PVD) and heterogeneity parameters (MFI heterogeneity, Het MFI). Briefly, to determine MFI, the image was divided into four quadrants and the predominant type of flow is assessed in each quadrant and characterized as absent = 0, intermittent = 1, sluggish = 2, or normal = 3. Values of the 4 quadrants were averaged. MFI heterogeneity was calculated as Het MFI = (MFI max − MFI min) × 100/MFI mean. For TVD and PVD, a gridline consisting of 3 horizontal and 3 vertical equidistant lines was superimposed on the image. All vessels crossing the lines were counted and classified either as perfused (continuous flow) or nonperfused (no flow or intermittent flow) vessels. Next, densities were calculated as the total number of vessels (TVD) or the number of perfused vessels (PVD), divided by the total length of the gridline in millimeters. PPV was calculated as PVD/TVD × 100 [17].

## 4. Statistical Analysis

In order to accomplish our objectives, patients evolving with versus without hyperlactatemia were compared for differences in severity scores, organ dysfunctions, hemodynamic and perfusion parameters, microcirculatory abnormalities, and hospital mortality.

Numerical variables were compared using Mann-Whitney *U* test, and categorical variables were compared by chi-square goodness-of-fit test. Spearman's correlation was used for testing between continuous variables, due to nonnormal distribution of data. Logistic and multivariate regression was performed to determine variables independently associated with hyperlactatemia, microcirculatory abnormalities, and hospital mortality. SPSS software version 17.0 (Chicago, IL, USA) was used for statistical calculations. Results are expressed as percentages or median and interquartile range. A *P* < 0.05 was considered as statistically significant. All reported *P* values are two sided.

## 5. Results

A total of 124 patients were included in this study. The general characteristics of the cohort are shown in Table 1. Thirty-eight patients (31%) did not present hyperlactatemia during resuscitation and 86 (69%) did. Sepsis was caused more frequently by abdominal and respiratory sources. Surgical resolution of sepsis foci was necessary in 39%.

When comparing both subgroups, no difference in comorbidities was found (Table 1). Patients without hyperlactatemia presented lower severity scores, less MV requirements, and lower hospital mortality (Table 1). They also exhibited higher platelet counts and lower serum creatinine levels (Table 2).

In relation to hemodynamic and perfusion parameters, patients with persistent sepsis-induced hypotension without hyperlactatemia presented lower NE requirements, less positive fluid balances, and received dobutamine less frequently (Table 3). A pulmonary artery catheter was inserted in 9 patients without hyperlactatemia and in 38 with elevated lactate levels. No significant differences in cardiac index, pulmonary artery occlusion pressure, ScvO_2_, and SvO_2_ were observed.

A sublingual microcirculatory assessment was performed in 45 patients (36% of the whole cohort; see above), 14 without and 31 with hyperlactemia. This subset was comparable to the whole cohort in clinical, hemodynamic, and perfusion variables, and outcome. When comparing subgroups, patients without hyperlactatemia exhibited significantly higher PPV and MFI values (Table 4).

In the subset of patients in whom a sublingual microcirculatory assessment was performed, lactate levels exhibited a significant correlation with PPV (Spearman's Rho = 0.499, *P* < 0.0001) and MFI (Spearman's Rho = 0.497, *P* < 0.0001).

## 6. Discussion

Our results confirm that patients with persistent sepsis-induced hypotension without hyperlactatemia present a very low mortality risk. This condition is associated with less organ dysfunctions and intensity of ICU management. Age, comorbidities, sepsis source control, and macrohemodynamic parameters including cardiac output, were not related to the presence or absence of hyperlactatemia. Interestingly, patients without hyperlactatemia presented less severe microcirculatory abnormalities and higher platelet counts. Although our conclusions are to some extent speculative and basically hypothesis generating, these data support the notion that patients with persistent sepsis-induced hypotension without hyperlactatemia exhibit a distinctive clinical and physiological profile.

Sepsis involves a complex interaction between the coagulation and inflammatory systems at the endothelial and microvascular level [18, 19]. This may result in tissue hypoperfusion, thus inducing hypoxia-driven hyperlactatemia [20]. Moreover, disseminated intravascular platelet activation may occur, contributing to microvascular failure and organ dysfunction [21]. Thrombocytopenia is a marker of this process. On the other hand, several microcirculatory abnormalities, such as endothelial edema, leukocyte activation, red blood cells stiffness, platelet aggregation, and functional shunting, could also induce microvascular hypoperfusion and eventually hyperlactatemia [22].

In effect, patients without hyperlactatemia evolved with higher platelet counts, a trend to lower D-dimer levels (*P* = 0.08), and a relatively preserved microcirculatory flow (PPV and MFI). Taken together, these data suggest that the absence of hyperlactatemia could be related, at least in part, to less severe endothelial and microcirculatory dysfunctions. As a matter of fact, macrohemodynamic variables, oxygen-derived parameters such as SvO_2_, and venous-arterial pCO_2_ gradients were not different between subgroups, thus suggesting that systemic flow disturbances are not major determinants of the genesis of hyperlactatemia in this setting.

The relationship between hyperlactatemia and microcirculatory abnormalities in septic patients is somehow controversial. Three studies reported a poor correlation between MFI and hyperlactatemia after single assessments [23–25]. In contrast, De Backer et al., testing the effect of dobutamine on microcirculatory abnormalities, found that an improvement in PPV was significantly associated with a decrease in lactate levels [26]. The same group confirmed these findings in another study addressing the effects of fluids on microvascular flow [27]. These discrepancies could be better explained by different study designs, concerning timing and number of microcirculatory assessments and therapeutic interventions. As a matter of fact, the latter group [26, 27] performed 2 sequential microcirculatory evaluations, thus comparing the time course of microvascular flow recovery and lactate decrease. In our case, although we performed a single microcirculatory assessment per patient, the main difference with the studies cited above [23–25] is that we compared microcirculatory derangements between two mutually exclusive subgroups and found a significant correlation between several microcirculatory flow-related parameters and lactate. Although methodological differences preclude a direct comparison between studies, in our opinion they ultimately suggest that there is an effective association between hyperlactatemia and microcirculatory abnormalities, at least during the early stages of septic shock. However, no definite cause-effect relationship can be established at this point.

 Another interesting finding is the relatively moderate degree of microcirculatory derangements found in our study, as shown by a mean MFI of 2.1 and a PPV of 75.5% in patients with hyperlactatemia. However, while our observation is consistent with recent studies that found similar mean basal MFI values [28–30], it is in sharp contrast with another trial reporting MFI values of less than 1.5 early after emergency room admission [31]. Moreover, Boerma et al. [32] reported that MFI improved over time (from 1.4 to 2.2) during resuscitation in the placebo arm of their nitroglycerin trial. These data considered together suggest that MFI values are very low in nonresuscitated patients but may improve rapidly after initial aggressive resuscitative maneuvers, resembling what happens with ScvO_2_. Nevertheless, this fact does not invalidate our results, since both subgroups, with and without hyperlactatemia, presented similar pre-ICU management and time from diagnosis to ICU admission (data not shown). Therefore, we believe that the observed differences in microcirculatory flow indexes are relevant and provide interesting potential clinical and physiological implications.

Our study suggests that persistent sepsis-induced hypotension without hyperlactatemia, traditionally included under septic shock definitions, constitutes a different subgroup in terms of prognosis and endothelial/microcirculatory dysfunction. Remarkably, more than 90% of these patients had this condition resolved and were discharged from ICU without further complications. Moreover, they required less intensive critical care treatment. The 2001 Sepsis Definition Conference proposed vasopressor requirements as a mandatory criterion for septic shock diagnosis, irrespective of lactate levels [7]. In this sense, besides confirming our previous retrospective findings [9], the present study provides more clinical and physiological data for a potential reappraisal of current septic shock definitions. The question whether persistent sepsis-induced hypotension without hyperlactemia constitutes a different pathophysiological entity, or simply a mild form of septic shock, should be addressed in future studies.

Our study has several limitations. This was a single-centre study, thus limiting the extrapolation of our results. Microcirculatory assessments were performed at different time points during early resuscitation, were limited to a subset of patients, and did not include serial measurements. We did not evaluate other potential mechanisms involved in the genesis of hyperlactatemia, such as hyperadrenergia with accelerated glycolysis, hepatosplanchnic flow, or mitochondrial dysfunction. No sample size calculation was performed, and our cohort was relatively small. We cannot rule out the possibility of having missed some high lactate values between sampling, although this is unlikely considering the frequent sampling. Finally, it was beyond our scope to comprehensively address all the potential causes of persistent hyperlactatemia. As stated in the introduction, this has been matter of extensive recent research, but briefly many potential nonhypoxic causes could contribute including hepatosplanchnic hypoperfusion, liver dysfunction, adrenergic-driven aerobic glycolysis, hyperinflammation, among others [1–3]. Nevertheless, we think that these results provide valuable information concerning the clinical and physiological significance of the absence of hyperlactatemia during sepsis-related circulatory dysfunction.

## 7. Conclusions

Persistent sepsis-induced hypotension without hyperlactatemia is associated with less severe organ dysfunctions and a very low mortality risk. Systemic flow parameters are not related to the presence or absence of hyperlactatemia. Our data suggest a relationship between coagulation, microcirculatory derangements, and lactate levels. This study tends to support the notion that patients with persistent sepsis-induced hypotension without hyperlactatemia exhibit a distinctive clinical and physiological profile within the spectrum of septic shock. This subject should be addressed in future studies.

## Figures and Tables

**Table 1:** General characteristics of the cohort and subgroups of patients.

	Total	Lactate < 2.5	Lactate ≥ 2.5
Number of patients	124	38	86
Age (y)	65 [53–75]	62 [39–73]	65 [58–75]
ICU LOS (d)	5 [3–9]	4.5 [2–7]	5 [3–10]
APACHE II score	18 [12–24]	12 [8–19]	20 [15–25]**
Basal SOFA score	8 [5–10]	6 [3–8]	9 [6–11]**
ICU mortality (%)	13.7	5.2	17.4*
Hospital mortality (%)	17.6	7.9	20.9*
Patients in MV (%)	79	71	82*
Length of MV (d)	2 [1–5]	1 [0–3.7]	3 [1–7]*
Renal replacement therapy	19	3	16*
Sepsis source (%)			
Pulmonary	27	26	28
Abdominal	45	45	44
Other	28	29	28
Adequate initial AB empiric coverage (%)			
Yes	81	71	85
No	13	16	12
Unknown	6	13	3
Comorbidities (%)			
Diabetes	20	19	21
Hypertension	26	23	27
Chronic kidney disease	7	6	8
Stroke	24	0	3
Atrial fibrillation	11	0	15

*P < 0.05 for the comparison between subgroups.

**P < 0.01 for the comparison between subgroups.

Data are shown as median [interquartile range] or percentage. ICU: intensive care unit; LOS: length of stay; APACHE: acute physiology and chronic health evaluation; SOFA: sequential organ failure assessment; MV: mechanical ventilation; AB: antibiotic.

**Table 2:** Baseline and peak laboratory parameters of organ dysfunction.

	Lactate < 2.5 mmol/l	Lactate ≥ 2.5 mmol/l
Baseline PaO_2_/FiO_2_	260 [185–388]	275 [160–339]
Lowest PaO_2_/FiO_2_	257 [184–340]	218 [150–286]
Baseline D-dimer levels (ng/mL)	3070 [2031–4198]	3788 [2096–5480]
Peak D-dimer levels (ng/mL)	3447 [2182–4771]	5298 [2885–7392]
Baseline platelet count (×10^3^/mm^3^)	192 [157–332]	145 [101–255]*
Lowest platelet count (×10^3^/mm^3^)	171 [116–261]	83.5 [43.3–162.5]**
Baseline bilirubin levels (mg/dL)	0.7 [0.5–1.3]	1 [0.6–1.9]
Peak bilirubin levels (mg/dl)	0.7 [0.6–1.7]	1.1 [0.7–3]
Baseline C-reactive protein levels (mg/dL)	15.9 [8.5–25.9]	14.7 [5.7–27.6]
Peak C-reactive protein levels (mg/dL)	24.4 [15.2–33.9]	28 [19.7–36]
Baseline serum creatinine levels (mg/dL)	0.8 [0.6–1.6]	1.7 [1–3]**
Peak serum creatinine levels (mg/dL)	1 [0.6–1.7]	1.7 [1.1–2.9]**

*P < 0.05.

**P < 0.01.

Data are shown as median [interquartile range].

**Table 3:** Hemodynamic and perfusion parameters in subgroups of patients.

	Lactate < 2.5 mmol/L	Lactate ≥ 2.5 mmol/L
Peak lactate level (mmol/l)	1.7 [1.3–2]	4.5 [3.4–7.4]**
Baseline lactate levels (mmol/l)	1.2 [1–1.8]	4 [3–5.8]**
Baseline PAOP (mmHg)	18 [13–26.5]	19.5 [15.3–23.8]
Baseline CI (l/min/m^2^)	3.2 [1.9–3.5]	3 [2.4 –3.7]
Lowest CI (l/min/m^2^)	2 [1.9–3.2]	2.4 [2 –2.7]
Lowest ScvO_2_ (%)	67 [59–71]	66 [58 –72]
Lowest SvO_2_ (%)	69 [65 –74]	68 [61 –75]
Peak NE dose (ug/kg/min)	0.08 [0.04 – 0.17]	0.2 [0.07–0.53]**
NE use (h)	22 [11–41]	35 [17–69]*
24 h fluid balance (mL)	1903 [845–2835]	4000 [1973–5509]**
Cumulative 72 h fluid balance (mL)	2857 [1130–5264]	5978 [3674–9551]**
Dobutamine use (% of patients)	18	46**
Basal P(cv-a)CO_2 _(mmHg)	5.5 [3–8]	6.1 [4.7–8]
Peak intra-abdominal pressure (mmHg)	19 [12.5–24]	17 [15–19]

*P < 0.05.

**P < 0.01.

Data are shown as median [interquartile range] or percentage. PAOP: pulmonary artery occlusion pressure; CI: cardiac index; ScvO_2_: central venous oxygen saturation; SvO_2_: mixed venous oxygen saturation; NE: norepinephrine; P(cv-a)CO_2_: central venous-to-arterial PCO_2_ difference.

**Table 4:** Hemodynamic, perfusion and microcirculatory parameters in 45 patients evaluated with sublingual SDF videomicroscopy.

	Lactate < 2.5 mmol/L	Lactate ≥ 2.5 mmol/L
*n* (%)	14 (31%)	31 (69%)
NE (ug/kg/min)	0.2 [0.09–0.39]	0.48 [0.22–0.93]*
Lactate (mmol/l)	1.4 [1.2–2.1]	5.8 [3.9–8.4]*
ScvO_2_ (%)	73 [67–77]	71 [66–78]
TVD (n/mm)	12.9 [10.7–13.9]	12.9 [11.1–14.9]
PVD (n/mm)	10.5 [9.5–12]	10.1 [6.6–12.4]
PPV (%)	87.3 [81.6–90.6]	75.5 [60.9–86.4]*
MFI	2.44 [2.25–2.61]	2.11 [1.7–2.32]*
Het MFI	0.33 [0.18–0.49]	0.42 [0.27–0.72]

*P < 0.01

Data are shown as median [interquartile range] or percentage. NS: nonsignificant (*P* > 0.05). NE: norepinephrine; ScvO_2_: central venous oxygen saturation; P(cv-a)CO_2_: central venous-to-arterial PCO_2_ difference; TVD: total vascular density; PVD: perfused vascular density; PPV: percentage of perfused vessels; MFI: microvascular flow index; Het MFI: MFI heterogeneity.
